# Risk Factors Associated with Helminthic Intestinal Infection in Lurambi Subcounty, Kakamega, Kenya

**DOI:** 10.1155/2020/8810519

**Published:** 2020-12-30

**Authors:** Ruth W. Kiiti, Elizabeth N. Omukunda, Jackson C. Korir

**Affiliations:** Department of Biological Sciences, Masinde Muliro University of Science and Technology, P.O. Box 190-50100, Kakamega, Kenya

## Abstract

**Background:**

Soil-transmitted helminths (STHs) and schistosome infections have been known to be major causes of morbidity and mortality in sub-Saharan countries. School aged and preschool children are known to be at high risk of infection. Therefore, the aim of this study was to determine the prevalence and risk factors associated with soil-transmitted helminths among school-going children in Lurambi Subcounty, Kakamega, Kenya.

**Method:**

A cross-sectional study was conducted from Jan 2020 to Feb 2020 among 392 randomly selected primary school-going children aged 5 to 14 years old in six primary schools. Risk factors associated with STH infection were obtained using a structured questionnaire answered by the children's caregivers. Stool samples were examined using the standard quantitative Kato-Katz technique. The data was analyzed using SPSS version 20 and Epi Info version 7.2.3.1.

**Result:**

A total of 278 children provided stool samples for analysis. The overall prevalence of intestinal helminths was 14.4% (40/278). The prevalence of *Ascaris lumbricoides* was 11.5% (32/278), 0.4% (1/278) for hookworm, 0.4% (1/278) for *Trichuris trichiura*, and 2.1% (6/278) for *Schistosoma mansoni*; coinfection was detected in 0.4% (1/278). The intensity of infection ranged between light and moderate. Significant risk factors for STH infection included failure to wash hands before eating (OR: 3.529; *P* = 0.041), failure to wash fruits and vegetables before eating (OR: 2.3129; *P* = 0.005), and not washing hands after soil contact (OR: 2.1529; *P* = 0.005). Age (*Z* = 2.4006, *P* = 0.0164) was a risk factor only for infection with *Schistosoma mansoni*.

**Conclusion:**

Preventive chemotherapy and proper hygienic and sanitation practices should be integrated to achieve elimination of STH and *Schistosoma mansoni* in Lurambi Subcounty and at large in Kenya.

## 1. Introduction

Soil-transmitted helminths (STHs) are parasitic nematode worms, which include *Ascaris lumbricoides*, *Trichuris trichiura*, *Strongyloides stercoralis*, and the hookworms, *Necator americanus* and *Ancylostoma duodenale*. Soil-transmitted helminth infections occur worldwide and are most commonly found in tropical and subtropical areas where sanitation and hygiene are poor [1]. These factors are found and are predominant in most developing countries, including Kenya, especially in rural areas which are at higher risk, and this implies that health awareness is needed. STHs survive well in warm and moist soil in tropical and subtropical countries [2]. A report by Pullan et al. [3] calculates that after interventions towards controlling STH were adopted, there are still approximately 1.45 billion people infected with soil-transmitted helminthiases in the world. STHs cause morbidity through blood loss leading to iron deficiency anemia, loss of appetite, competition for micronutrients, malabsorption of nutrients [4], and suppression of the immune system; this leads to retarded growth in the child , low development, and poor educational performance [5]. Approximately 10 million people in Kenya are infected with STH, and more than 12 million Kenyans inhabiting rural endemic areas are at risk of infection [6], including school-age children (SAC) [7]. Risk factors such as sociodemographic and socioeconomic characteristics in Lurambi Subcounty, Kakamega, Kenya, might be playing a great role in the spread and transmission of soil-transmitted helminths among school-going children. The prevalence of STH is high within Kakamega County with ranges from 26.7% to 62.6% for *A. lumbricoides* and low for hookworm (1.8%), with Kakamega central having the highest prevalence within the county (52.1%) despite continuous deworming since 2014 [7]. Intestinal schistosomiasis is caused by *Schistosoma* species (*S. guineensis*, *S. intercalatum*, *S. mansoni*, *S. japonicum*, and *S. mekongi*); the parasite infects humans by penetrating through the skin when man comes into contact with water that is infested with water that is infested with schistosome cercariae [1]. Intestinal schistosomiasis can cause abdominal pain, diarrhoea, and blood in the stool. Liver enlargement is common in advanced cases and is frequently associated with an accumulation of fluid in the peritoneal cavity and hypertension of the abdominal blood vessels; in such cases, there may also be enlargement of the spleen [8]. It is estimated that 240 million people worldwide have been affected by schistosomiasis and 700 million people live in regions known to be endemic areas, with most of the cases occurring in sub-Saharan Africa [9]. The World Health Organization (WHO) highlighted the need for repeated mass drug administration (MDA) on a population at high risk at regular intervals in order to bring down schistosomiasis infection [1, 10]. The predominant causative agent of intestinal schistosomiasis in Kenya is *Schistosoma mansoni* [11]. In Kenya, it is estimated that 6 million people are infected with *S. mansoni*, with more than 15 million people at risk of infection [8]. As a result of the negative effects of worms on the health and education of children, the Ministry of Health and the Ministry of Education launched a National School-Based Deworming Programme (NSBDP) in 2009 [12]. The mass deworming treatment was administered to school-going children, and preschool-going children, including those out of school. This was performed with the aim of reducing the prevalence of STH and schistosome infections to below 1% [11, 13]. The national control team incorporation with county and subcounty levels works together in the administration of albendazole for STHs and praziquantel for schistosomiasis. However, although continuous treatment for STHs has been administered, treatment for schistosome infections has been interrupted due to lack of praziquantel [14]. The current study was conducted in order to provide information that may be used by government and stakeholders in child health care institutions, primary schools, and the community institutions for the development of strategic control programs that can operate alongside the current deworming programs to achieve maximum control and prevention of soil-transmitted helminthiasis infection and schistosomiasis in the county and the country at large.

## 2. Materials and Methods

### 2.1. Study Area

**Figure 1:**
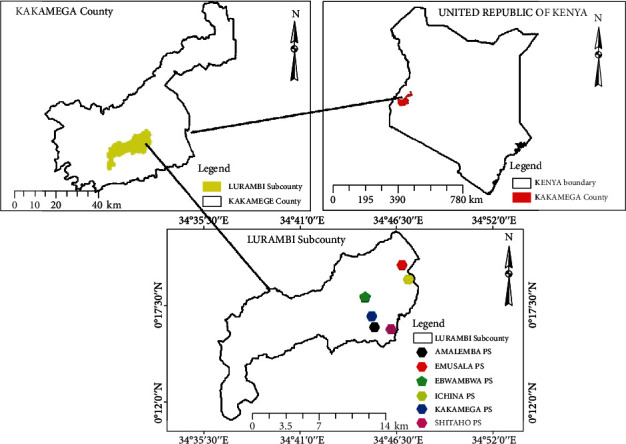
A map showing the study area.

The study was conducted in Lurambi Subcounty, found in Kakamega County, Kenya (Figure 1), which has an approximated population of 160,229 people covering an area of 161.8 km^2^. Lurambi Subcounty has a total of 161 primary schools. Its wards include Butsotso East, Butsotso South, Butsotso Central, Shieywe, Mahiakalo, and Shirere. They practice intensive farming of maize, tea, beans, and horticultural crops on a small scale. The annual rainfall range in the county is from 1280.1 mm to 2214.1 mm per year.

### 2.2. Study Population

The study population entailed of pupils aged between 5 and 14 years from Lurambi Subcounty primary schools whose caregivers gave consent to the study. Those pupils who were below 5 years and above 14 years old, pupils whose caregivers did not give consent, and those pupils who were not able to give fresh stool were excluded from the study,

### 2.3. Sample Size

Fisher formula [15] was used to determine the desired sample size. The sample size allowed the estimation of the prevalence of soil-transmitted helminth (STH) infection.

(1) $$\begin{array}{c} \left(n\right)=\frac{\left(Z^{2}P_{q}\right)}{d^{2}}, \end{array}$$

where (*n*) was the required sample size, *Z* is the *Z* value for a given confidence level, *p* is the expected prevalence *q* = (1 − *p*)and *d* is the allowable error of estimation. The confidence level was assumed to be 95% with an allowable error of 5%, and thus *Z* was 1.96. Prevalence of STH among school-going children in Kakamega County was assumed as 50% and was used in obtaining the sample size. The minimum sample size was 392 school-going children.

### 2.4. Sampling Procedure

The study utilized a cluster approach, which was defined as schools due to homogeneity. Based on the central limit theorem, the number of clusters was determined by dividing the sample size (*n* = 392) with the minimum acceptable sample per cluster (66 pupils), yielding 6 clusters that formed the study sampling frame (Table 1). The study adopted a 2-stage sampling procedure from the selection of schools to pupils as follows: level 1: a list of primary schools in Lurambi Subcounty, Kakamega County, was obtained from the District Education Office. Six (6) out of all primary schools within Lurambi Subcounty, Kakamega County. Three schools were selected randomly from the following areas: rural and urban; level 2: a registration list of pupils (aged 5–14 years) from the selected schools was obtained from the head teacher's office. The simple random sampling method using the registration list was employed to select pupils from each school using a table of random numbers. A request note was sent to the parents through their children, inviting them to school on the days agreed on with the principal investigator. Upon the parents' turnout in the school, the principal investigator explained to them about the study in a confidential manner. The issue of informed consent, risks and benefits, voluntariness, confidentiality, and procedure of the study were explained to the caregivers of the children. Thereafter, the parents were requested to consent on behalf of their children and to respond to questionnaires administered.

### 2.5. Parasitological Examination

The pupils were guided on how to provide 2 grams of fresh stool sample. Every child who had been selected in the sampling frame provided a fresh stool sample on two consecutive days. The fresh stool samples were immediately transported in a cool box maintained at temperature < 10°C to Masinde Muliro University of Science and Technology Zoology Laboratory for analysis. The Kato-Katz standard method was used to determine the presence of egg and counts of soil-transmitted helminths in defined quantities [16]. Two Kato-Katz slides per stool were prepared using the fixed quantity of sieved 41.7 mg (WHO kit) of stool on a punched template. Then, it was mounted on slides and covered with malachite green impregnated cellophane. The slides were observed within one hour under the microscope at a magnification of ×40. The slides were observed immediately for hookworm while for *Ascaris lumbricoides*, *Trichuris trichiura*, and *Schistosoma* species, eggs were examined 60 minutes later. Quality control was performed by a systematic random examination in the schools while collecting the stool sample, and the slides that were randomly drawn were examined by qualified and trained personnel following the standard operating procedures for the Kato-Katz technique and through double reading of the slides by a qualified microscopist.

### 2.6. Data Analysis

The data were entered and analyzed using SPSS version 20 and Epi Info version 7.2.3.1. Prevalence of helminthic infections was calculated using descriptive statistics. The association between helminthic infection and risk factors was determined by multivariate logistic regression at 95% confidence interval. Any association was significant when the *P* value was <0.2 during the univariate analysis and for it to qualify for multivariate analysis, and finally, a risk factor was statistically significantly associated with any helminthic infection when the *P* value < 0.05. The intensity of infection for intestinal helminths was calculated by multiplying the number of observed eggs of each helminth by 24 to give eggs per gram of faeces (EPG).

## 3. Results

### 3.1. Prevalence

A total of 392 school-going children were enrolled for the study; 392 (100%) parents gave consent for their children while 114 (29.1%) of the children included in the study were unable to give a stool sample. Out of the 278 participants who gave the stool sample, the number of females was slightly higher (51.8%) than male participants (48.2%); this was not statistically significant. Fifty-four percent of the children whose parents gave consent were aged between 5 years and 10 years, and 54.0% of the caregivers had attained only primary school education. Forty point six percent (40.6%) of the caregivers were self-employed, and 63.7% of the participants were enrolled in rural primary schools.

The overall prevalence of STH and schistosome infection was 14.4%. Males showed a higher prevalence (7.6%) than girls (6.8%), but the difference was not statistically significant (Table 2). The overall prevalence was highest in children between the ages of 5 and 10 years, concretely, 10.1% (Table 2). The prevalence of infected children whose caregivers were unemployed also showed the highest prevalence (10.4%) with the lowest prevalence found in those children whose parents were formally employed (0.4%). Washing of hands with only water after soil contact and after using the toilet as a control practice showed the highest prevalence of infection, with 12.6% and 11.9%, respectively. Children whose parents had primary school education level were mostly infected with STH and schistosomes with a prevalence of 12.2%. Schools located in the rural regions had a prevalence of 11.9% (Table 2).

### 3.2. Risk Factors Associated with Helminthic Intestinal Infection

The failure to practice washing of hands before eating by school-going children showed the highest likelihood of getting infected with soil-transmitted helminths with an odds ratio of 114.47 (CI: 10.44-1255.62; *P* = 0.0001), and this was statistically significant. Occupation level of caregiver, failure to wash hands after using the toilet, place of swimming, education level of the caregiver, and washing of hands after soil contact also recorded high risk in the transmission of soil-transmitted helminths. The failure to wash hands after coming into contact with soil showed high odds ratio, indicating that children who did not wash their hands with water and soap had a likelihood of 9.81 (CI: 1.66-58.02; *P* = 0.01) times more of being infected with soil-transmitted helminths than those who wash their hands with water and soap after soil contact, and this was also statistically significant. The risk factors, which include practices such as failure to wash fruits and vegetables before eating, had an odds ratio of 1.36 (CI: 0.45-4.15). However, it was statistically insignificant (*P* = 0.5906). This factor showed that the number of children who do not practice washing of fruits and vegetables before eating had a likelihood of 1.36 times more of being infected with soil-transmitted helminths than children who washed fruits and vegetables before eating. The child gender, age group, and shoe wearing habit had a regression coefficient below zero indicating that the risk factor assessed was a preventive factor. This is because shoe wearing was encouraged in all primary schools, so a significant proportion of the children were wearing shoes, and the odds ratio of these factors was below one, which indicated that the factor was preventive.

Children who did not practice washing hands between handling raw and cooked food were 4.91 (OR: 4.91; CI: 0.66-36.33) times more likely to be infected with soil-transmitted helminths than those who washed their hands though statistically insignificant (*P* = 0.1191). School-going children who were not washing their hands using soap and water after visiting the toilet were 29.80 more likely to be infected with soil-transmitted helminths (OR: 29.80 CI: 3.71-239.36). Those children who were not practicing washing of hands with soap and water were 114.47 times more likely to be infected with soil-transmitted helminths (OR: 114.47; CI: 10.44-1255.62).

School going children who were swimming in rivers were 13.13 times more likely to be infected with soil-transmitted helminths and *Schistosoma mansoni* (OR: 13.13; CI: 1.76-98.17), and this was statically significant (*P* = 0.0014). The use of night soil (use of faeces as fertilizer) was 2.10 times more likely to contribute to infection with soil-transmitted helminths (OR: 2.10; CI: 0.41-10.78), and this was not statistically significant (*P* = 0.3755). Those children who go to rural primary schools were 4.97 times more likely to be diagnosed with soil-transmitted helminths and *Schistosoma mansoni* (OR: 4.97; CI: 0.72-34.42), and this was not statistically significant (*P* = 0.1041). The habit of sharing bathroom materials like towels was a risk factor. Children who were from families where they shared bathroom materials were 4.57 times more likely to be infected with soil-transmitted helminths and *Schistosoma mansoni* (OR: 4.57; CI: 0.61-34.14) though statistically insignificant (*P* = 0.1383). It was also found that school-going children whose caregivers were not formally employed and self-employed were 34.21 times more likely to be infected with soil-transmitted helminths and *Schistosoma mansoni* (OR: 34.21; CI: 3.25-360.72) which was statistically significant (*P* = 0.0033). The education level of caregivers of the children was also a risk factor with odds of 11.10 more likely to have their children infected with soil-transmitted helminths (OR: 11.10; CI: 0.95-130.05) though statistically insignificant (*P* = 0.0553) (Table 3).

## 4. Discussion

In this study, the overall prevalence of STH was significantly lower as compared to what was seen in a study carried out in informal urban schools by Ngonjo et al. [7] in Kakamega Central. This is in tandem with Mwandawiro et al.'s [11] survey in selected counties within Kenya, which showed a significant reduction of STH and schistosomes after repeated observation from 2012 to 2017. *Ascaris lumbricoides* was the most predominant etiological agent in this assessment which agrees with other researchers [7, 17, 18]; however, it disagrees with Halliday et al. [19] and Loukouri et al. [18] who recorded lower prevalence of *A. lumbricoides* in South coastal Kenya. Sang et al. [20] recorded a lower prevalence of *A. lumbricoides* in their assessment at South Nyanza Kisumu. The predominance of *A. lumbricoides* can be attributed to its high rate of reinfection when compared to other soil-transmitted helminths, which are supported by the observations in other studies [11, 21, 22]. In their studies, they found out that school-going children could easily be reinfected by *A. lumbricoides* after being subjected to anthelminthic treatment and because of poor personal hygiene practiced by the children in Lurambi Subcounty, removing shoes after reaching school, and the climate in the region which is warm and moist which favours the growth and survival of the *A. lumbricoides*. Also, its eggs can remain dormant and withstand harsh adverse conditions in the soil for up to 10 years [23].

Hookworm was detected in this study but with a low prevalence (0.4%), which can be transmitted within the subcounty if control measures are not practiced, which differs with Ngonjo et al. [7] who did not encounter any hookworm in the Kakamega Central. The prevalence of *T. trichiura* was also low, which is different from the prevalence observed in South Nyanza [20], and this agrees with another study by Loukouri et al. [18] in Eastern Cote d'Ivoire. Multiple infections between *A. lumbricoides* and hookworm were observed in this study which agrees with other studies [24–27] in which they observed multiple infections that are polyparasitism, coinfection of two STH species, and coinfection with three soil-transmitted species. Shitaho Primary School accounted for the highest prevalence of STH, and this is in tandem with Ngonjo et al. [7]. However, an appreciable reduction of the prevalence was observed in this study. The overall prevalence of *S. mansoni* was 2.1%, similar to what was observed by Mwandawiro et al. [11]. This study records a lower prevalence of *S. mansoni* than that recorded in Kisumu (13% prevalence) [20]. School-going children aged 11-14 years had higher prevalence compared to those aged 10 years and below, and this is in line with Sang et al. [20]. Sex of the children did not statistically influence the prevalence of the STH and *S. mansoni*, which agrees with findings from previous studies [7, 28, 29]. Preventive chemotherapy alongside observation and control of potential risk factors is the most effective way of avoiding reinfection and drug resistance. Ranjan et al. [29] reported that continuous deworming should be integrated with the observation of hygienic practices both at school and community levels in order to be able to achieve elimination of these intestinal worms. In this study, failure to wash hands before eating, after using the toilet, and after soil contact; wearing of shoe behaviour; occupation of the caregiver; and eating unwashed fruits and vegetables were the risk factors which were statistically significant to soil-transmitted helminths and *S. mansoni*.

The caregiver education level is a significant factor in controlling STH infection as it was ranked as a risk factor in this study, which agrees with Naish et al. [30] where caregiver education was related to infection intensity of STH. The occupation level was statistically significant to STH and *S. mansoni* infection, and this was also observed by Naish et al. [30]. This study noted that eating unwashed fruits and vegetables was attributed to STH infection, which agrees with findings from previous studies [27, 31]. The observation made can be explained by what was observed by Bekele et al. [32] and Tefera et al. [33] in their study on parasitic contamination of raw vegetables and fruits, where they concluded that the fruits and vegetables were contaminated with STH. Place of swimming was also associated with STH and *S. mansoni*, and this agrees with Damen et al. [34]. Children going to rural schools had high odds of being infected with STH and *S. mansoni* compared to those going to urban schools. This observation is similar to what was observed by Ranjan et al. [29] where children who were from periurban areas had 1.53 times higher odds of being infected with the intestinal worms than those from urban areas. Shoe wearing was observed as a preventive factor associated with STH and *S. mansoni*, which agrees with Halliday et al. [19]. The infection intensity of *S. mansoni* was associated with a risk factor of swimming in rivers which was observed in other studies [35, 36] which concluded that the intensity of infection with *Schistosoma* species is influenced by the time spent in invaded water and continuous contact with water invaded with schistosomes. The age of the child was statistically significant to *S. mansoni* intensity (*P* = 0.0164) which agrees with Sang et al. [20].

This study has several limitations. First, the Kato-Katz technique used was not able to detect *Strongyloides* species. Second, the study sample size was determined using an estimated prevalence. Third, the number of samples that were analyzed for the prevalence of STH and *Schistosoma* species became smaller than the expected due to failure of some study participants to provide a fresh stool sample for the two consecutive days. Fourth, the study did not focus on factors such as the intensity of intestinal helminths in soil, community, and distribution of snail species and *Schistosoma* infection among snails which are crucial aspects for STH and Schistosomiasis control.

## 5. Conclusion

The study carried out indicated the risk factors associated with helminthic infection within Lurambi Subcounty, Kakamega, and therefore, we encourage the integration of preventive chemotherapy and proper hygienic and sanitation practices in order to eliminate STH and *Schistosoma mansoni* in Lurambi Subcounty and at large in the whole country, Kenya.

## Figures and Tables

**Table 1:** Sample size and study population.

School	Description	Boys	Girls	Subtotal
Kakamega primary	Urban setting	33	33	66
Amalemba primary	Urban setting	33	33	66
Ebambwa primary	Urban setting	33	33	66
Ichina primary	Rural setting	33	33	66
Emusala primary	Rural setting	33	33	66
Shitaho primary	Rural setting	33	33	66

**Table 2:** Prevalence of intestinal worms in Lurambi Subcounty in Kakamega, Kenya, obtained using chi-square in SPSS version 20.

		Intestinal helminths			
		Absence		Presence	
		Count	*N*%	Count	*N*%
Sex	Male	113	40.6%	21	7.6%
	Female	125	45.0%	19	6.8%
Occupation	Farming	62	22.3%	5	1.8%
	Self-employed	108	38.8%	5	1.8%
	Formal employment	41	14.7%	1	0.4%
	Unemployed	27	9.7%	29	10.4%
Education level of caregiver	Informal	20	7.2%	5	1.8%
	Primary	116	41.7%	34	12.2%
	Secondary/above	102	36.7%	1	0.4%
Source of food	Cooked from home	220	79.1%	40	14.4%
	From hotel	6	2.2%	0	0.0%
	Home and hotel	12	4.3%	0	0.0%
	Others	0	0.0%	0	0.0%
Eating of raw fruits & vegetable	Yes, frequently	115	41.4%	32	11.6%
	Yes, sometimes	103	37.1%	4	1.4%
	No	20	7.2%	4	1.4%
Wash fruits & vegetables	Yes, always	89	32.0%	5	1.8%
	Yes, occasionally	124	44.6%	29	10.4%
	No	25	9.0%	6	2.2%
Eat undercooked raw meat	Yes, always	0	0.0%	0	0.0%
	Yes, occasionally	0	0.0%	0	0.0%
	No	238	85.6%	40	14.4%
Eat undercooked raw fish	Yes, frequently	0	0.0%	0	0.0%
	Yes, occasionally	0	0.0%	0	0.0%
	No	238	85.6%	40	14.4%
Wash hands after soil contact	Yes, with water and soap	86	30.9%	0	0.0%
	Yes, with only water	132	47.5%	35	12.6%
	No	20	7.2%	5	1.8%
Wash hands between handling raw and cooked food	Yes, with water and soap	60	21.6%	0	0.0%
	Yes, with only water	164	59.0%	34	12.2%
	No	14	5.0%	6	2.2%
Wash hands before eating	Yes, with water and soap	69	24.8%	1	0.4%
	Yes, with only water	165	59.4%	35	12.6%
	No	4	1.4%	4	1.4%
Wash hands after visiting toilet	Yes, with water and soap	84	30.2%	2	0.7%
	Yes, with only water	138	49.6%	33	11.9%
	No	16	5.8%	5	1.8%
Wash hands after touching toilet materials	Yes, with water and soap	76	27.3%	2	0.7%
	Yes, with only water	142	51.1%	33	11.9%
	No	20	7.2%	5	1.8%
Source of water for washing clothes and utensils	Pipe water	88	31.7%	5	1.8%
	River	111	39.9%	35	12.6%
	Spring/well	39	14.0%	0	0.0%
	Rainwater	0	0.0%	0	0.0%
Source of water for bathing	Pipe water	90	32.4%	5	1.8%
	River	109	39.2%	35	12.6%
	Spring/well	39	14.0%	0	0.0%
	Rainwater	0	0.0%	0	0.0%
Source of drinking water	Pipe water	97	34.9%	5	1.8%
	River	91	32.7%	35	12.6%
	Spring/well	50	18.0%	0	0.0%
	Rainwater	0	0.0%	0	0.0%
Water treatment	Yes	149	53.6%	21	7.6%
	No	89	32.0%	19	6.8%
Method of water treatment	No treatment	88	31.7%	17	6.1%
	Boiling	72	25.9%	20	7.2%
	Filtering	37	13.3%	1	0.4%
	Commercial tablets/liquids	41	14.7%	2	0.7%
Place of swimming	No swimming	119	42.8%	6	2.2%
	River	81	29.1%	34	12.2%
	Lake	2	.7%	0	0.0%
	Swimming pool	36	12.9%	0	0.0%
Shoe wearing habits	Yes, always	101	36.3%	31	11.2%
	Yes, occasionally	128	46.0%	9	3.2%
	No	9	3.2%	0	0.0%
Ownership of latrine	Yes	226	81.3%	40	14.4%
	No	12	4.3%	0	0.0%
Usage of latrine	Private for household	136	48.9%	37	13.3%
	Shared with neighbours	102	36.7%	3	1.1%
Usage of night soil	Yes	81	29.1%	30	10.8%
	No	157	56.5%	10	3.6%
Usage of toilet material	Privately	68	24.5%	9	3.2%
	Common for the family	170	61.2%	31	11.2%
Diarrhoea last 3 months	Yes	73	26.3%	33	11.9%
	No	165	59.4%	7	2.5%
Time of last diarrhoea	Did not have diarrhoea	166	59.7%	7	2.5%
	Before a month	21	7.6%	4	1.4%
	Before two weeks	25	9.0%	29	10.4%
	Within two weeks	26	9.4%	0	0.0%
Went to hospital	Yes	51	18.3%	1	0.4%
	No	187	67.3%	39	14.0%
Presence of intestinal worms	Yes	21	7.6%	0	0.0%
	No	217	78.1%	40	14.4%
Taking antiparasitic drugs	Yes	193	69.4%	36	12.9%
	No	45	16.2%	4	1.4%
School setup	Urban school	94	33.8%	7	2.5%
	Rural school	144	51.8%	33	11.9%
Age group distribution	5-10 years old	122	43.9%	28	10.1%
	11+ years old	116	41.7%	12	4.3%

**Table 3:** The multivariate analysis of risk factor and the presence of intestinal worm.

Term	Odds ratio	95%	C.I.	Coefficient	S. E.	*Z*-statistic	*P* value
Sharing of the bathroom material	4.57	0.61	34.14	1.52	1.03	1.48	0.1383
Age of child	1.06	0.72	1.57	0.06	0.20	0.29	0.7687
Age group	0.38	0.04	3.47	-0.98	1.13	-0.86	0.3889
Education level of caregiver	11.10	0.95	130.05	2.41	1.26	1.92	0.0553
Occupation level of caregiver	34.21	3.25	360.72	3.53	1.20	2.94	0.0033
School setup	4.97	0.72	34.42	1.60	0.99	1.63	0.1041
Use of night soil	2.10	0.41	10.78	0.74	0.84	0.89	0.3755
Sex of child	0.95	0.26	3.43	-0.06	0.66	-0.08	0.9331
Wearing of shoes	0.03	0.00	0.21	-3.50	0.98	-3.56	0.0004
Place swimming	13.13	1.76	98.17	2.58	1.03	2.51	0.0121
Washing fruits & vegetables	1.36	0.45	4.15	0.31	0.57	0.54	0.5906
Washing of hands after soil contact	9.81	1.66	58.02	2.28	0.91	2.52	0.0118
Wash hands between handling raw & cooked food	4.91	0.66	36.33	1.59	1.02	1.56	0.1191
Wash hands after using toilet	29.80	3.71	239.36	3.39	1.06	3.19	0.0014
Wash hands before eating	114.47	10.44	1255.62	4.74	1.22	3.88	0.0001

## Data Availability

Data are available from the corresponding author upon request.
